# Crafting Appealing Text Messages to Encourage Colorectal Cancer Screening Test Completion: A Qualitative Study

**DOI:** 10.2196/mhealth.4651

**Published:** 2015-11-04

**Authors:** Kathryn E Weaver, Shellie D Ellis, Nancy Denizard-Thompson, Donna Kronner, David P Miller

**Affiliations:** ^1^ Wake Forest School of Medicine Department of Social Sciences and Health Policy Winston-Salem, NC United States; ^2^ University of Kansas School of Medicine Department of Health Policy and Management Kansas City, MO United States; ^3^ Wake Forest School of Medicine Department of Internal Medicine Winston-Salem, NC United States

**Keywords:** colorectal cancer, screening, mhealth, text messages, qualitative, older adults

## Abstract

**Background:**

mHealth interventions that incorporate text messages have great potential to increase receipt of preventive health services such as colorectal cancer screening. However, little is known about older adult perspectives regarding the receipt of text messages from their health care providers.

**Objective:**

To assess whether older adults would value and access text messages from their physician’s practice regarding colorectal cancer screening.

**Methods:**

We conducted four focus groups with 26 adults, aged 50 to 75 years, who had either recently completed or were overdue for colorectal cancer screening. A trained moderator followed a semistructured interview guide covering participant knowledge and attitudes regarding colorectal cancer screening, potential barriers to colorectal cancer screening, attitudes about receiving electronic communications from a doctor’s office, and reactions to sample text messages.

**Results:**

Participant responses to three primary research questions were examined: (1) facilitators and barriers to colorectal cancer screening, (2) attitudes toward receiving text messages from providers, and (3) characteristics of appealing text messages. Two themes related to facilitators of colorectal cancer screening were perceived benefits/need and family experiences and encouragement. Themes related to barriers included unpleasantness, discomfort, knowledge gaps, fear of complications, and system factors. Four themes emerged regarding receipt of text messages from health care providers: (1) comfort and familiarity with technology, (2) privacy concerns/potential for errors, (3) impact on patient-provider relationship, and (4) perceived helpfulness. Many participants expressed initial reluctance to receiving text messages but responded favorably when shown sample messages. Participants preferred messages that contained content that was important to them and were positive and reassuring, personalized, and friendly to novice texters (eg, avoided the use of texting shorthand phrases and complicated replies); they did not want messages that contain bad news or test results. They wanted the ability to choose alternative options such as email or phone calls.

**Conclusions:**

Older adults are receptive to receiving cancer screening text messages from health care providers. Sharing sample messages with patients may increase acceptance of this tool in the clinic setting. Supportive tailored text messaging reminders could enhance uptake of colorectal cancer screening by enhancing patient self-efficacy and providing cues to action to complete colonoscopy or fecal occult blood testing.

## Introduction

Over the last decade, mobile phone ownership has increased dramatically in the United States. Approximately 90% of US adults now own a mobile phone, and 58% own a smartphone with access to the Internet [1]. This high prevalence of mobile phone ownership is similar across racial and rural/urban categories [1]. Even among individuals with low incomes and little education, mobile phone ownership exceeds 84% [1]. In contrast, a significant digital divide exists for home broadband Internet access where large differences are seen along racial, economic, educational, and geographic lines [2].

The popularity of mobile phones affords an opportunity to reach broadly across populations, including members of medically underserved communities. Over 80% of mobile phone owners use their devices to send or receive text messages, representing a low-cost method of communication [3]. Several studies have demonstrated that text messaging interventions can have positive effects on weight loss, physical activity, disease self-management, smoking cessation, medication adherence, and appointment attendance [4-8]. However, the majority of these studies have targeted younger populations.

Many diseases disproportionally affect older adults. As an example, colorectal cancer, which is the second leading cause of cancer deaths in the United States, predominately occurs in adults over age 50 [9,10]. Routine screening can prevent colorectal cancer and decrease mortality, yet approximately one-third of Americans fail to receive regular screening [11].

Text messaging interventions could encourage screening and support patients throughout the screening process. However, it is unclear how agreeable older patients would be to receiving colorectal cancer screening-related text messages. In studies conducted in older patients with heart disease in New Zealand, most participants were interested in receiving cardiac rehabilitation text messages and when sent messages, 82% read some or all of them [12,13]. In contrast, two other studies found that less than half of older patients opted for text messages [14,15]. In addition, recent systematic reviews conclude that more research is needed to define the optimal content and structure of text messaging interventions [7].

To assess whether older adults would value and access text messages from their physician’s practice, we conducted a qualitative study examining the potential utility of text messages to support colorectal cancer screening. We particularly wanted to explore older adults’ thoughts about receiving health-related text messages and their opinions regarding the characteristics of desirable messages. This new information is needed to guide the development of text messages that are acceptable and useful to an older population.

## Methods

### Participants

We recruited participants from three community-based primary care practices affiliated with a large academic medical center in Winston-Salem, North Carolina, selected to represent a diverse patient population in urban, suburban, and rural locations. The urban practice serves a primarily socioeconomically disadvantaged patient population; the other two practices serve mostly insured patients. The Wake Forest Baptist Health Institutional Review Board approved the study protocol. All participants provided written informed consent and received a $25 gift card for participating in a focus group (held November-December 2013).

To identify potential participants, we queried electronic medical records to identify patients aged 50 to 74 years who had completed a primary care visit within the last 6 months. We mailed recruitment letters to 300 patients with no documented history of colonoscopy within the last 10 years and 161 patients with a documented colonoscopy within the last 3 years. Patients were invited to call to enroll in the study, and a study staff member also made follow-up calls to invite participation. We formed four focus groups of 6 to 10 participants each (2 focus groups from the urban practice and 1 focus group each from the suburban and rural practices). As groups were formed, we selectively called patients to ensure a balance of gender, age (50-64 years vs 65-74 years), screening status, and use of technology (experience with text messaging vs no experience).

### Baseline Data Collection

All participants completed a telephone survey assessing mobile phone ownership, text messaging frequency, health literacy level, and history of colorectal cancer screening. Health literacy was measured with the screening item, “How confident are you filling out medical forms by yourself?” [16]. Participants were categorized as adequate (quite a bit or extremely) or low (somewhat, a little bit, or not at all) literacy. To contextualize the analysis, we used information from the initial telephone survey questionnaire combined with demographic information (education, health insurance coverage) collected in a brief on-site questionnaire.

### Focus Group Structure

**Figure 1:**
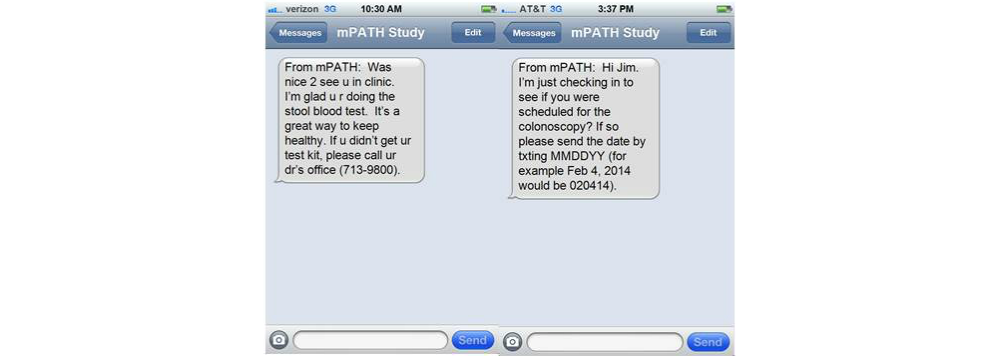
Examples of text messages that participants viewed regarding colon cancer screening.

A trained moderator led each focus group using a semistructured moderator guide, which was revised as needed after each focus group to validate concepts and subsequent investigator interpretations emerging from previous groups. Each focus group explored participant knowledge, attitudes, and beliefs about colorectal cancer screening; potential barriers to colorectal cancer screening; attitudes about receiving electronic communications from a doctor’s office; and reactions to a sample of text messages about colorectal cancer screening designed to vary by use of language, formality, and requests for a reply. To prime the discussion, each group watched a 6-minute video colorectal cancer screening decision aid during the focus group encounter. During the course of the focus groups, participants were sent sample text messages regarding colorectal cancer screening on their personal mobile phones or were shown sample text messages on a large screen (see Figure 1). The messages varied in the formality (eg, use of first names vs last names), length, use of shorthand text phrases, and interactivity (ie, instruction to text back a date or a “1” to confirm receipt). All focus groups were audio-recorded, transcribed, and de-identified to protect participant confidentiality.

### Analysis

We conducted a descriptive thematic content analysis [17]. Interim analysis revealed that saturation had been reached, with no new themes arising from the data [18]. To ensure consistency in coding, we created a preliminary coding dictionary based on themes explored in the moderator guide [17-19]. After coding a sample of the transcripts, we added codes based on group consensus of themes not captured by the preliminary coding scheme. Using this final coding dictionary, each focus group transcript was then coded by one investigator and reviewed by a second investigator for agreement. Disagreements were resolved by group consensus. Four investigators (KW, ND, SE, and DM) each served as a primary coder and secondary coder for one transcript.

## Results

### Overview

We conducted four focus groups (26 participants), each lasting 90 minutes. Characteristics of participants are shown in Table 1. In accordance with our purposive sampling strategy, 27% of our participants reported not being up to date on colorectal cancer screening, and 42% reported being regular users of text messaging. We analyzed emergent themes guided by three primary research questions: (1) facilitators and barriers to colorectal cancer screening, (2) attitudes toward receiving text messages from providers, and (3) characteristics of appealing text messages.

**Table 1:** Characteristics of focus group participants.

Demographic characteristics		N=26
Age, years, mean (range)		60 (50-73)
Female, n (%)		16 (62)
**Race/ethnicity, n (%)**		
	White, non-Hispanic	10 (38)
	African-American	16 (62)
**Educational attainment, n (%)**		
	Less than high school	3 (12)
	High school graduate	10 (38)
	More than high school	13 (50)
Covered by health insurance, n (%)		19 (73)
Up-to-date on colorectal cancer screening, n (%)		19 (73)
Low health literacy, n (%)		8 (31)
Uses text messaging regularly, n (%)		11 (42)

### Facilitators to Colorectal Cancer Screening

Quotations illustrating the seven themes regarding facilitators and barriers of colorectal cancer screening are shown in Textbox 1. Participants perceived benefits associated with early detection and the need to be checked.

When you have cancer or any other kind of illness, that's a lotta times there's nothing that you, yourself, can do about it. But if you go…and get some help, then it's a good chance that everything will be all right.

adequate literacy, screened female

Many participants described screening as a very matter of fact decision. Patients often mentioned that their doctor had recommended screening when they turned 50. Others mentioned prompting by media sources such as a physician television personality. Finally, some participants reported that symptoms, most commonly blood in the stool, motivated them to be tested.

**Representative quotes for primary barriers and facilitators of colorectal cancer screening by emergent theme.**

Facilitators:

- Perceived benefit/need: benefit of early detection of colorectal cancer and need to be checked for cancer.

I reckon for health, I didn't want anything to go wrong there, and I just went in and took care of it.

adequate literacy male

- Family experiences and encouragement: diagnosis or screening of a family member or direct prompting or encouragement by a family member.

She (my wife) got me to go down and do it and I’m glad I did now.

adequate literacy male

Barriers:

- Unpleasantness: concern about the bowel preparation process or handling stool sample.

I hate the prep. I just can’t, there's gotta be an easier way than drinking all that liquid, and just feeling like you're blown up.

low literacy male

- Discomfort: actual or anticipated discomfort associated with colorectal cancer screening, typically colonoscopy.

I won’t go back unless I'm guaranteed to be knocked out. …I said, ‘I may actually end up with colon cancer because I am terrified of the screening.’ It was horrible.

adequate literacy female

- Knowledge gaps: lack of knowledge or misinformation about colorectal cancer and screening guidelines.

Well, I just told him no, I didn't wanna do it, you know? I didn't see any—there was nothing wrong with me.

adequate literacy female

- Fear of complications: concern regarding actual or anticipated complications.

I know one of my members, they had it, and they was sick for days.

low literacy female

- System factors: difficulties with appointments, referrals, or insurance or use of reminder letters or calls.

Sometimes at the doctor's office, for you to get…a referral from a doctor's office, that's pulling teeth.

low literacy female

Family experiences with colorectal cancer diagnosis and treatment were described by several screened participants. Others mentioned direct prompting by family members as an important factor in their decision to be screened or described encouraging other family members to be tested.

### Barriers to Colorectal Cancer Screening

Barriers associated with unpleasantness and discomfort were commonly mentioned by participants, especially with regards to colonoscopy. The bowel cleaning preparation was frequently described as unpleasant (eg, taste, volume of beverage required, nausea, need to be in close proximity to bathroom), but for most screened participants it did not appear to be an actual barrier to a repeat procedure. Anticipation of unpleasantness was perceived as a barrier for others.

I think that's the biggest hang-up about having a colonoscopy. People are so afraid of the prep. It truly is not so bad anymore.

adequate literacy female

Concerns about discomfort during the colonoscopy were also mentioned, but most people reported that it was painless. Multiple participants reported a desire to be sedated or “knocked out.” However, for two screened women, a prior painful colonoscopy was a significant barrier to repeat screening. Adequate literacy participants appeared more likely to mention unpleasantness, but there were no demographic differences for concerns about discomfort.

Knowledge gaps were a barrier to screening for at least some participants, particularly those without health insurance. These gaps manifested as misperceptions (only men need it, screening is only needed if you have symptoms) and questions that arose during the focus group regarding correct screening interval, accuracy of different tests, difference between sigmoidoscopy and colonoscopy, and desire for more information about polyps. Knowledge-related barriers did not appear to differ by health literacy, education, or clinic location.

Some participants also mentioned fear of complications (ie, anesthesia or the endoscopy damaging something). A few participants mentioned experiencing complications or suspected complications (eg, hemorrhoids); others mentioned friends or family members with more serious complications following the procedure (eg, hospitalization or infection). This appeared more common among adequate literacy participants.

Finally, a few participants, most but not all of whom were adequate literacy and insured, noted that efficient office systems could increase colorectal cancer adherence. Others reported problems associated with obtaining a referral and scheduling the appointment (system factors). For some it was like “pulling teeth” [low literacy female].

### Attitudes Toward Receiving Text Messages From a Health Care Provider

Four key themes emerged regarding text messages from health care providers: (1) comfort and familiarity with technology, (2) privacy concerns and potential for errors, (3) impact on patient-provider relationship, and (4) perceived helpfulness. The overall positive or negative valence of expressed attitudes did not appear to vary by setting (urban, underserved practice vs suburban/rural practice). Quotations illustrating each theme are shown in Textbox 2.

**Representative quotes regarding text messages from a health care provider by emergent theme.**

- Comfort and familiarity: comfort with use of text messaging and other new communication strategies (mixed valence).

It seem like now, since I'm over 50 now, I notice, they kinda pushing me into this technology and it's like this is not me.

adequate literacy female

Well, I get texts from my doctor, from my dentist, especially, send texts all the time.… I've been doing it now for a couple of years, and it's fine with me.

adequate literacy female

- Privacy concerns/potential for errors: concern about others accessing personal information or mix-ups (negative valence).

If it was a doctor doing it—which I know it can't be him—he would have my files right there in front of him…. But if you get other people doing it, I think that's where the problem will come in that could be errors, and that would make me a little bit scared.

low literacy female

- Impact on patient-provider relationship: positive ability to enhance communication and sense of caring or negative perception of technology as impersonal (mixed valence.

Doctors especially in a clinic like this see so many people—and you don't have a really tight relationship with your doctor anymore it seems. And a text just makes it that much more impersonal.

adequate literacy male

I just feel that it would be nice of him if he told me to get it and I didn't get it, to remind me that I didn't get it, and he hasn't got the results back yet. So I think that's caring on his part if you receive texts.

low literacy female

- Perceived helpfulness: sense that information would be helpful or redundant with current communication (mixed valence).

People say, ‘I didn’t get no paper in the mail,’ saying something happened with the mail or you forget. It’s good to get reminders.

adequate literacy female

I just don't see how it could possibly be feasible for doctors to be sending out this kind of stuff. And if they have done their job while you were there, this shouldn't be necessary. All of that should have been covered.

adequate literacy female

Participants varied in their comfort and familiarity with text messaging, with some expressing positive attitudes regarding its ease and convenience or prior experiences with text reminders from other providers such as dentists or patient portals. Convenience was discussed slightly more among those with adequate health literacy and those who had health insurance compared to those with low health literacy and those with no insurance. More commonly, participants expressed concern regarding lack of familiarity with text messaging or perceptions that it was impersonal, intrusive, or for a younger generation.

Now if you call my house and leave a message on my answering machine, I will reply to you, I will do it. And I think that's a little bit more personal, you know. I've always said text is for people that don't want to talk to you. And this is the generation we're in now. They don't have time to sit down and talk on the phone.

adequate literacy female

A number of participants with adequate health literacy were not regular text message users, and general concerns about technology were more commonly expressed by non-texters. Expressed texting familiarity did not seem to be associated with health insurance status.

Several participants also expressed concerns about privacy or the potential for errors when text messaging with providers. There was a perception that text messages might not be secure; about half of participants expressed concerns about other people accessing personal information, either by accessing a phone or through more generalized security breaches. Participants with adequate health literacy and those with health insurance were more likely to discuss these concerns than those with low health literacy and those with no health insurance. Other participants expressed concerns about mix-ups or errors (wrong patient or wrong test results) or intentional deception from someone “playing a joke.” These types of concerns appeared more common among participants with adequate health literacy and those with health insurance. Participants who were not regular texters also raised concerns about errors more often than those who were regular texters.

Participants expressed diverse opinions about the impact of text messaging on their relationship with their provider. Some participants felt texts were impersonal and a poor substitute for face-to-face communication, but others perceived them as caring or supportive. Concerns about the impact on patient-provider relationship were expressed slightly more often among those who were not habitual texters than those who did text as a means of communication.

Participants also expressed divergent opinions about the perceived helpfulness of text messages from health care providers. Some participants viewed reminders as indicative that a medical office failed to properly inform the patient at the time of the initial appointment or that a patient was not taking sufficient responsibility. These participants believed there was no need for such messages.

I know what I need to do. I appreciate you reminding me of my appointment, but I'm a grown man. If I'm gonna make this appointment, I'm gonna make it. If I'm not, nothing you say is gonna make me make it….just call and remind me of—if need be—of my appointment. That's that.

low literacy male

Others acknowledged that reminders are helpful and that the information contained in text messages might be helpful for later reference. There were no apparent differences in attitudes regarding helpfulness by participant characteristics.

### Characteristics of Appealing Text Messages

Participants responded much more positively when viewing sample text messages (see Figure 1) than when asked about general opinions regarding text messages. Feedback about the specific text messages or text messaging in general did not differ by health literacy, health insurance coverage, education, or regular use of text messaging. Characteristics of appealing text messages are summarized in Textbox 3.

**Suggestions for crafting appealing text messages from medical providers.**

- Message should have a positive, reassuring tone (eg, “Hang in there, Bob”); informal messages are commonly perceived as more positive.

- Text messages should be personalized to provide reassurance that it is going to the right person; individuals vary in their preference for use of first or last names.
- The message should be tailored so that it contains content that is important to the person, recognizing that individuals disagree about what type of content is important (eg, appointment reminders).
- Texts should be friendly to novice texters:
  - Avoid use of texting shorthand phrases such as “nice 2 see u.”
  - Messages should not require participants to text back dates or other information; participants were more comfortable with short numerical replies to confirm dates or receipt (eg, “Text 1 to confirm”).
- Content should not contain bad news or test results.
- Patients should have alternatives (opt out of text messages and/or receive letters or phone calls if they prefer).

Participants generally found the messages easy to comprehend and potentially helpful or encouraging.

Yeah, it's kind of encouraging—the prep may be a little challenging, but it'll be okay

adequate literacy female

Participants wanted a positive, reassuring tone. Less formal language was perceived as somewhat more supportive and caring. For example, several participants responded very positively to this text message: “Hang in there, Jim. I know the prep and clear diet can be challenging, but it is well worth it. Remember you will need to have someone to take you to the test tomorrow.”

Although many responded positively to the overall content, some disliked the use of the first name. Personalization was deemed very important because it confirmed the message was going to the intended recipient.

The content of the messages was perceived as very important, but participants differed in their opinions about what content was necessary. There was a desire for messages to be brief but informative. Some participants viewed the doctor’s phone number or reminders about tests as unnecessary, but others thought it was helpful or caring. Other participants expressed a preference for only getting a brief text message to call their doctor or did not want the word “colonoscopy” to be used in the message.

Participants wanted text messages that were friendly to novice texters. Participants did not like use of shorthand text abbreviations (eg, nice 2 see u) and felt it could be confusing. Many participants had limited comfort with interactivity, preferring simple instructions like “Text 1 to confirm that you received this message” over instructions to text back numerical dates.

With regards to general thoughts regarding the use of the text messages in the health care setting, there was strong consensus that the content of text messages should not contain bad news or test results.

Many participants also wanted alternative options like phone, letter, or email or the choice to opt out of all messages.

So now instead of automatically sending you a message like that, also, ask, ‘Do you want a reminder?’ …I don't need that reminder

adequate literacy female

It's a personal thing, and you have to approach people in the way they feel most comfortable

adequate literacy female

Preferred mode of communication did not appear to differ based on health literacy, education, health insurance, or use of texting. Cost of text messaging was a concern expressed by only one participant.

## Discussion

### Principal Findings

In four focus groups of diverse individuals with varying colorectal cancer screening histories, we explored barriers and facilitators to colorectal cancer screening and identified several suggestions for developing text messaging interventions targeting older individuals. Our results confirmed many of the previously reported colorectal cancer screening facilitators (perceived benefits, family history, and encouragement) and barriers (disdain for the bowel prep and fear of procedural complications) [20-23]. We identified several themes relating to positive and negative attitudes about receiving text messages from a health care provider that may be important to consider when designing text messaging interventions and systems. Although many participants in our study had limited familiarity with text messaging and initially expressed some concerns, they were generally positive when shown sample text messages pertaining to screening. From their feedback about our sample text messages, we derived several recommendations about crafting appealing health-related text messages. Carefully designed text messages have the potential to overcome some of the identified barriers to colorectal cancer screening by providing encouragement and reassurance and enhancing self-efficacy to complete colorectal cancer screening tests.

### Comparison With Prior Work

This qualitative study is one of the first to explore older adult attitudes about receiving cancer screening text messages. A few studies have examined the use of text messages for care along the cancer continuum [24]. Two survey studies found that 37% to 45% of women were agreeable to receiving mammogram reminders by text message [25,26], and a pilot study of cervical cancer screening text messages reported high satisfaction with the intervention [27]. We are unaware of prior studies investigating willingness to receive text messages encouraging colorectal cancer screening, a topic many adults find distasteful [28]. Other researchers have reported variability in older adults’ willingness to receive health-related text messages [13-15]. The increased receptivity we observed after participants viewed sample messages suggests that sharing some potential messages with participants may increase program uptake. In addition, studies of text message reminders have generally found high levels of patient satisfaction [8].

### Implications for Design of Text Messages

A major goal of our study was to determine what content and characteristics would make a text message appealing or objectionable to older adults. Systematic reviews of text message interventions have specifically called for more research in this area [7]. We derived several key recommendations for crafting appealing health-related text messages (summarized in Textbox 3). Focus group participants wanted messages that were positive, tailored so that content was relevant, and personalized (with name) and did not contain bad news or test results. In addition, the messages should not use texting shorthand phrases or require participants to text back anything more than a simple, single character reply. Supporting these recommendations, a meta-analysis of text message interventions for health promotion found that personalized and tailored messages were associated with greater efficacy [29]. While we are not aware of prior studies specifically examining older adults’ attitudes about the specific content and format of text messages, qualitative studies in adolescents and young adults reinforces the importance of texts using casual, supportive language and containing helpful information [30-33].

Participants in our study also wanted the choice to opt out of text messages and receive the health information by email or some other means. This finding is consistent with studies finding varied preferences for receiving automatic reminders [14,15,34-37]. Younger age has been associated with an increased preference for text messages over phone calls or emails [15,36]; however, individuals’ familiarity with sending or receiving text messages is a greater predictor of patient acceptance [15,37]. Given that adult use of text messaging has increased dramatically over the last decade [38,39], more and more people will likely choose texting as their preferred communication modality in the future.

A distinct advantage of text messages is the ability to easily tailor them to the individual. Our results reveal some tailoring may be required to craft text messages that are broadly appealing, but this should be achievable with relatively simple algorithms (eg, patient preference for first or last name). With tailoring, potential differences in preferences based on literacy, gender, and mode of screening can be accommodated. Appealing clinic reminder systems will allow participants to choose greeting (first or last name), level of detail, and modality (text, email, or phone).

### Implications for Colon Cancer Screening Practice and Research

Discussions about colonoscopy dominated all the focus groups, with few people expressing knowledge of other screening tests. Others have similarly reported that patients are more knowledgeable about colonoscopy than fecal screening tests [22,40]. However, when patients are informed of their screening options, almost as many will chose fecal occult blood testing as will chose colonoscopy [41-43]. Given the colonoscopy-specific barriers we and others have observed, offering fecal testing as an alternative has the potential to increase screening rates.

Future research should investigate which colorectal cancer screening messages are most effective and whether offering a variety of delivery modalities (such as email vs text messaging) increases efficacy. Based on our findings, our research team has created a series of messages to support fecal occult blood testing and colonoscopy for colorectal cancer screening. We are currently testing these messages in a multisite randomized controlled trial, and we let participants choose between email and text message delivery.

### Limitations

This study adds substantially to the literature on mHealth interventions by including the perspectives of patients who are not regular users of text messaging and patients with low health literacy. Limitations of our study relevant to the generalization of results include the predominant focus on colorectal cancer screening and the exclusion of younger and older adults who would not be eligible for colorectal cancer screening. In addition, although we broached concepts derived from earlier groups with subsequent groups to assess their salience and our interpretation of these findings, we did not provide full results of our findings back to the participants for validation [19].

### Conclusions

Our results have several implications for encouraging colorectal cancer screening and the use of text messages more broadly in clinical practice. Participants were generally more positive when they viewed sample text messages. It may be important to show patients examples of the messages they will receive to increase buy-in; tailoring and personalization are also key. In summary, we believe texting can be a powerful tool to address health disparities through its ability to connect with hard to reach populations to encourage completion of preventive health services such as colorectal cancer screening.
